# Evaluation of the safety profile and intrapulmonary pharmacokinetics of intravenous fosfomycin in healthy adults

**DOI:** 10.1128/aac.01395-24

**Published:** 2025-01-08

**Authors:** Lindsay Boole, Zhonghui Yang, Stephen P. Bergin, Robert M. Tighe, Emily Randolph, Byron Hauser, Kenan Gu, Varduhi Ghazaryan, Alison Wall, Katherine Weigand, Emmanuel B. Walter, Loretta G. Que

**Affiliations:** 1 Division of Pulmonary, Allergy, and Critical Care Medicine, Duke University School of Medicine 12277 , Durham, North Carolina, USA; 2 Duke Human Vaccine Institute, Duke University School of Medicine 12277 , Durham, North Carolina, USA; 3 Division of Microbiology and Infectious Diseases, National Institute of Allergy and Infectious Diseases, National Institutes of Health 540429 , Bethesda, Maryland, USA; 4 Emmes Company 6947 , Rockville, Maryland, USA; 5 Department of Pediatrics, Duke University School of Medicine 12277 , Durham, North Carolina, USA; Providence Portland Medical Center, Portland, Oregon, USA

**Keywords:** fosfomycin, bronchoscopy, intrapulmonary pharmacokinetics, healthy participants

## Abstract

This Phase 1 trial described the intrapulmonary pharmacokinetics and safety profile of IV fosfomycin in healthy participants*.* Fosfomycin, a broad-spectrum antibiotic mainly used to treat urinary tract infections, is being considered for treatment of more complex conditions, including lung infections, due to the emergence of multidrug-resistant (MDR) organisms. Despite its potential, the pharmacokinetics and safety profile of intravenous (IV) fosfomycin, particularly its penetration into the lower respiratory tract, are unknown. To address this gap, we conducted a Phase 1, open-label trial to assess the safety and pulmonary pharmacokinetics of IV fosfomycin in healthy participants. Thirty-seven healthy volunteers aged 18–45 years received three doses of 6 g IV fosfomycin every 8 hours. Bronchoscopy with bronchoalveolar lavage (BAL) was performed at randomly assigned time points after the third dose. BAL fluid, BAL cell pellets, and blood plasma samples for fosfomycin were analyzed using validated assays of liquid chromatography with tandem mass spectrometry (LC-MS/MS). Adverse events (AEs) were assessed. Fosfomycin exhibited penetration into alveolar macrophages (AM) at a rate of 16.8% and into the extracellular lining fluid (ELF) at 30.8%. Mean AM fosfomycin concentration ranged from 14.8 to 32 μg/mL, while the mean ELF concentration ranged from 15.7 to 82.5 μg/mL. All participants experienced at least one treatment-emergent adverse event (TEAE), mostly mild/grade 1, with no serious adverse events (SAEs) reported. Intravenous fosfomycin effectively penetrates both the extracellular (ELF) and intracellular (AM) compartments of the lower respiratory tract in healthy participants. The overall tolerability of IV fosfomycin was favorable, suggesting its potential as an effective antibacterial treatment for lower respiratory tract infections.

**CLINICAL TRIALS:**

This study is registered with ClinicalTrials.gov as NCT03910673.

## INTRODUCTION

Bacterial resistance against antimicrobials has reached alarming levels in recent decades, resulting in crisis and necessitating the need for more antibiotic treatment options, particularly among Gram-negative bacteria, including those that express extended-spectrum beta-lactamases (ESBLs) and the carbapenem-resistant Enterobacteriaceae (CRE). The lack of available and effective antibiotics for these organisms has created an unmet medical need that is widely acknowledged (1). Infections with CRE are difficult to treat, and treatment options are limited or unsuitable for certain patients. Mortality rates as high as 40%–50% have been associated with CRE infection, and thus the Centers for Disease Control and Prevention (CDC) considers this an urgent threat to public health (2).

Fosfomycin exhibits bactericidal activity against anaerobic pathogens and many Gram-positive and -negative bacteria, including the increasingly problematic CRE- and ESBL-producing organisms. Currently, oral fosfomycin is FDA-approved in the United States for treatment of urinary tract infections (UTIs). In Europe and Japan, for over 45 years, IV fosfomycin has been a safe and effective option for treating patients with UTIs and a variety of other, often very severe, infections, including MDR infections in patients with cystic fibrosis (3) and sepsis (4). IV fosfomycin penetrates rapidly into tissues and achieves clinically relevant concentrations in serum, urine, soft tissues, lung, bone, cerebrospinal fluid, and heart valves, making it a desirable antibiotic treatment option (5). Despite decades of use, fosfomycin has retained excellent antimicrobial activity. The emergence of MDR organisms such as ESBL and CRE has prompted consideration of IV fosfomycin in the United States and Canada for broader sites of infection (6).

Recent studies have provided useful information for modernizing and optimizing fosfomycin dosing regimens. Total daily doses of 8 g and 32 g have been shown to result in a rapid reduction in the bacterial density without amplification of a drug-resistant subpopulation (7). The lowest fosfomycin dosing regimen found not to amplify a drug-resistant bacterial subpopulation, thus achieving a resistance-inhibitory concentration (RIC), was 4 g administered every 8 hours (8).

Two studies to-date of fosfomycin penetration into lung tissue evaluated single doses. Farago et al*.* evaluated pulmonary penetration of a single 2-g dose administered either IV or intramuscular (IM) 1 to 2 hours before planned pulmonary surgery. The penetration rate of fosfomycin into the total combined content of the resected lung tissue was 32%–52% of serum levels (9). Matzi *et al*. used microdialysis probes in the interstitial pulmonary space during pulmonary surgery to evaluate pulmonary interstitial penetration of a single 4-g IV dose in patients undergoing thoracotomy for treatment of complicated pneumonia, such as pulmonary abscess or empyema. The fosfomycin penetration rate into the pulmonary interstitial space 1 hour after the dose was 44%–55% of plasma levels. (4)

The safety of IV fosfomycin has previously been studied in the form of a single, variable dose (1 g; 8 g) in healthy adults and in comparison to piperacillin–tazobactam in adults with complicated UTI (10, 11). TEAEs in these prior studies have included mild bradycardia; asymptomatic laboratory abnormalities, including hypocalcemia, hypokalemia, and transaminase elevation; gastrointestinal side effects; and mild headache. The only SAE deemed related to fosfomycin in either prior trial was hypokalemia. (10)

This trial sought to further evaluate the safety of multiple 6-g doses of fosfomyin, a dosing regimen higher than the established RIC, and to characterize the pharmacokinetics (PK) of its penetration into the lower respiratory tract.

## MATERIALS AND METHODS

### Study design

We performed a Phase 1, open-label trial to evaluate the safety and pharmacokinetics, including in the plasma and lower respiratory tract, of three 6-g doses of IV fosfomycin in healthy participants. This study was conducted at the Duke University Early Phase Clinical Research Unit (DEPRU) after approval by the Duke University Health System Institional Review Board. This study was registered at clinicaltrials.gov as NCT03910673 and conducted in accordance with the principles of Good Clinical Practice and the Declaration of Helsinki.

### Study population

After obtaining written informed consent, healthy volunteers aged 18–45 years were screened, which included a complete medical history, ascertainment of use of all prescription and non-prescription drugs, alcohol and tobacco use, physical exam, electrocardiogram (ECG), laboratory tests for assessment of electrolytes, organ function, alcohol and tobacco status, screening for coronavirus disease 2019, and pregnancy status for females of childbearing potential. Healthy individuals with a body weight >50 kg and body mass index 18–30 kg/m^2^ without clinically significant findings on the screening and baseline evaluations were included. Complete inclusion and exclusion criteria are detailed in Table S1. Participants meeting eligibility criteria were confined at the DEPRU.

### Study procedures

After admission, enrollment, and baseline clinical and laboratory assessments, participants received fosfomycin 6 g IV every 8 hours for three doses. Blood samples for PK measurements were collected at established pre- and post-dose time points. For the first dose, samples were collected prior to dose start time, at 30 minutes after first dose start time (during the 1-hour infusion), at 1 hour, 1 hour 15 minutes, 2 hours, and 5 hours after the first dose start time. For the second dose, only a pre-dose blood sample was collected. For the third dose, blood samples were collected prior to the dose start time, at 30 minutes after the third dose start time (during the 1 hour infusion), at 1 hour, 1 hour 15 minutes, 2, 5, 8, and 12 hours. Within 30 minutes of collection, blood tubes were centrifuged at approximately 2,000 g for 15 minutes at 5°C. Plasma was extracted into cryovials, immediately placed on dry ice, and stored at –70°C or below.

Upon admission and prior to dosing, each participant was randomly assigned to a single time point for bronchoscopy with BAL sampling (Fig. S1). The BAL time points were 30 minutes, 1 hour 15 minutes, 2 hours, 5 hours, and 8 hours after start of the third dose of IV fosfomycin. The participants were assigned randomly to one of the five BAL time points, 1:1:1:1:1, using AdvantageEDC randomization software.

After local administration of lidocaine on the vocal cords, followed by moderate sedation, the bronchoscope was introduced into the bronchial tree, and serial lavage was performed with instillation of four 50-mL aliquots of sterile normal saline into the right middle lobe; return was achieved with gentle aspiration. The aspirates recovered from the second, third, and fourth instillations were pooled and placed on ice. After removal of 4 mL of BAL fluid for cell count and differential analyses, the remaining pooled BAL was centrifuged in one 50-mL tube at no more than 50 mL volume each time in a refrigerated centrifuge (Eppendorf 5702R) at 400 g for 5 minutes to separate the BAL supernatant containing ELF from the cell pellet containing mostly AM. After the first centrifugation of 50 mL BAL, the supernatant was separated and placed on ice. The remaining BAL fluid was poured into the same tube and was centrifuged under the same conditions. Only one centrifuge tube was used in order to combine the cell pellet. The BAL supernatant was aliquoted into four 5-mL tubes and several 10-mL tubes and stored at −70°C immediately. The cell pellet was re-suspended to a total volume of less than 250 µL of buffered saline (the resuspension volume was adjusted according to pellet size) and stored at −70°C. A blood sample for plasma urea concentration was obtained simultaneously to the BAL sampling time.

In addition to baseline assessments previously noted, safety evaluations, including symptom assessments, vital signs, physical exam, blood and urine laboratory assessments, and ECG, were conducted at time points throughout the dosing period and at discharge from inpatient confinement. A follow-up telephone call 3 days after the first study dose, following discharge from the DEPRU, was conducted to assess for the development and resolution of any adverse events.

Halting criteria included any two subjects with any related Grade 3 systemic AE, any two subjects with the same related Grade 3 laboratory AE, or any one subject with a related SAE. An independent safety monitor was established to independently evaluate any SAEs and Grade 3 related AEs, and a safety monitoring committee reviewed AE data throughout the study and was available to provide recommendations in the event that any halting criterion was met.

### Participant withdrawal and replacement

Participants were permitted to voluntarily withdraw from the study at any time, and investigators could withdraw participants for any reason, including, but not limited to, no longer meeting eligibility criteria; noncompliance or loss to follow-up; new clinical diagnosis or finding that could potentially compromise the safety of the study or interfere with assessments during the study; or pregnancy. In the event of participant withdrawal prior to receiving all three doses of IV fosfomycin and undergoing bronchoscopy with concurrent blood sampling, withdrawn participants were replaced by alternate participants. Participants who withdrew or were withdrawn following fosfomycin dosing were asked to continue in planned safety and AE assessments, including follow-up of any existing AEs to resolution or until deemed stable.

### Analysis of plasma, BAL supernatant, and AM concentrations of fosfomycin

Plasma, BAL supernatant, and AM samples were analyzed at an independent, central lab using validated LC-MS/MS bioanalytical assays to determine the fosfomycin concentration of fosmomycin. Assays measured total fosfomycin concentrations; fosfomycin is known to have negligible protein-binding (12). The assay for fosfomycin in human plasma had a range of 1.0 to 400 µg/mL. The within-run accuracy (%Bias) was −4.3 to 3.0% for the lower limit of quantitation (LLOQ), and the within-run precision (% coefficient of variation, %CV) was 3.7% to 7.4%. The range was extended to 1500 µg/mL with validated dilutional linearity testing. The assay for fosfomycin in human BAL supernatant had a range of 1.0 to 300 µg/mL. The %Bias was −8.3 to 4.0% for LLOQ, and the %CV was 2.5% to 8.7%. The range was extended to 1,130 µg/mL with validated dilutional linearity testing. The assay for fosfomycin in AM had a range of 1.0 to 300 µg/mL. The %Bias was −5.5 to 3.0% for the LLOQ, and the % CV was 6.2% to 11.5%. The range was extended to 1,130 µg/mL with validated dilutional linearity testing.

In the initial analysis of BAL supernatant samples, fosfomycin concentrations were below the LLOQ for 29 out of the 30 specimens due to a higher-than-expected degree of saline dilution from the BAL process. These samples were subsequently retested using a partially validated LC-MS/MS bioanalytical assay with an LLOQ of 0.1 µg/mL. The validated parameters included standard curve linearity, quality controls for accuracy and precision, carryover and freeze/thaw, and bench time stability.

### Analysis of plasma and BAL supernatant concentrations of urea

Urea was also measured using a validated LC-MS/MS assays in the BAL supernatant and in the plasma samples simultaneous to each BAL. The assay for urea in human plasma had a range of 10 to 1000 µg/mL. The %Bias was 4.0 to 16.0% for the LLOQ, and the %CV was 4.9% to 7.1%. The range was extended to 7,500 µg/mL with validated dilutional linearity testing. The assay for urea in the BAL supernatant had a range of 1 to 100 µg/mL. The %Bias was −8.7 to 3.0% for the LLOQ, and the % CV was 5.2% to 9.9%. The range was extended to 750 µg/mL with validated dilutional linearity testing.

### Calculation of ELF and AM concentrations of fosfomycin

Urea concentrations in plasma and BAL supernatant were adjusted for dilution of the ELF by the saline administered for the BAL, using the assumption that urea has equal concentrations in serum and ELF. (13, 14)

Fosfomycin concentration in ELF (C_fosfo,ELF_) was calculated after fosfomycin concentration in the BAL supernatant (C_fosfo,BAL_) and urea concentrations in the BAL supernatant (C_urea,BAL_) and in plasma (C_urea,plasma_) were measured. The following equations were used:


$$C_{fosfo,ELF}=C_{fosfo,BAL}\times (V_{BAL}/V_{ELF}),$$

where V_ELF_ and V_BAL_ are the volumes of ELF and BAL, respectively, derived by


$$V_{ELF}=V_{BAL}\times (C_{urea,plasma}\;/\;C_{urea,BAL}).$$

Thus,


$$C_{fosfo,ELF}=\left(C_{urea,plasma}\;/\;C_{urea,BAL}\right)\times C_{fosfo,BAL}.$$

Fosfomycin concentration in AM (C_fosfo, AM_) was calculated by


$$C_{fosfo,AM}=C_{pellet\;suspension}/V_{AM},$$

where C_pellet suspension_ is the amount of fosfomycin measured in the cell pellet suspension and V_AM_ is the volume of AM in the cell pellet suspension. V_AM_ was determined by multiplying the cell count in BAL fluid by the mean AM cell volume of 2.42 µL/10^6^ cells (14); thus,


$$V_{AM}=number\;of\;AM\;cells\;in\;BAL\;x\;2.42\;\mu L/10^{6}\;cells.$$

### Outcome measures

All participants who received any amount of IV fosfomycin were included in the analysis population for safety. The safety outcome measure was occurrence of AEs, including SAEs, at any time from the start of study drug administration through the end of participant follow-up. Incidence and frequency of AEs were determined.

The PK analysis was performed using the PK population of evaluable participants. An evaluable participant was defined as a participant who received all doses of IV fosfomycin, underwent bronchoscopy at the assigned sampling time point with BAL return volume adequate for sample analysis, and underwent at least one blood sampling time point concurrent with the BAL sampling time point, with blood sampling volume adequate for sample analysis.

A plasma PK profile was determined via creation of fosfomycin plasma concentration versus time curves following IV fosfomycin administration. Noncompartmental analysis was used to determine plasma PK parameters using Phoenix WinNonlin version 8.3 and using plasma concentrations collected after the start of the first and third doses.

To derive the intrapulmonary PK, the ratios of pulmonary ELF and AM concentrations of fosfomycin to simultaneous plasma concentrations were calculated for each participant and summarized for each sampling time. The median concentrations and geometric mean of fosfomycin from the plasma and BAL sampling times were used to estimate the population average area under the concentration versus time curve between 0 and 8 hours (AUC_0-8_) of plasma, ELF, and AM, and the AUC extrapolated to infinity (AUC_0-inf_) of plasma. The ratio of population average AUC_0-8_ of ELF-to-plasma and AM-to-plasma was calculated to determine the percent penetration into each substrate.

## RESULTS

### Population

**Fig 1:**
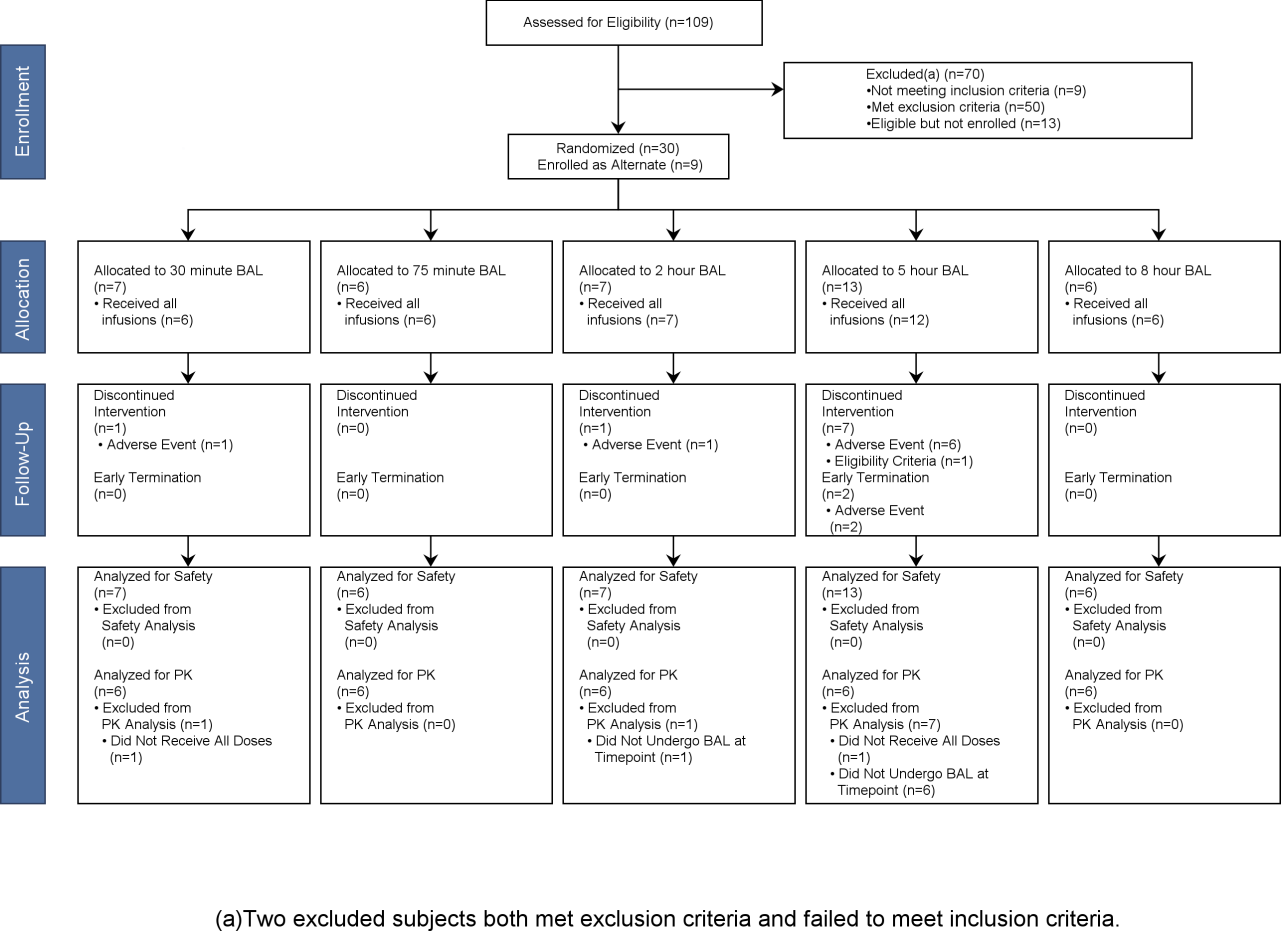
CONSORT diagram of subject disposition.

A total of 109 participants were screened, and 30 were randomized plus nine served as alternates. Of 59 participants who were excluded due to failure to meet inclusion/exclusion criteria, the most common reasons for screen-failure were body weight or body mass index (BMI) outside of acceptable range, one or more screening lab values outside of acceptable range, and liver disease. Six participants were eligible but elected not to enroll, citing the study time commitment was too high. Another five participants were eligible but elected not to enroll due to concerns for potential risks of study participation. Two participants were eligible but were ultimately excluded due to other reasons. Figure 1 details the disposition of all participants.

Demographic data are summarized in Table 1. There were similar numbers of male (54%) and female (46%) participants. The majority of participants were non-Hispanic or Latino (87%). Forty-one percent were White, 38% were Black, 13% were Asian, and 3% were American Indian. The mean age of participants was 31.4 years (range: 19 to 45 years) with a median age of 30.0 years. The mean height of participants was 168.89 cm (range: 156.0 to 189.5 cm) with a median height of 167.30 cm. The mean weight of participants was 72.84 kg (range: 50.5 to 94.3 kg) with a median weight of 72.50 kg. The mean BMI of participants was 25.48 kg/m^2^ (range: 18.8 to 30.4 kg/m^2^) with a median BMI of 25.30 kg/m^2^. Age, height, weight, and BMI were similar across the assigned BAL sampling timepoints. The most common pre-existing health conditions across all participants were history of surgical or medical procedures (38%) and eye disorders (31%). Eighteen percent of participants reported use of concurrent medications, with the majority being related to the alimentary tract and metabolism (three participants; 8%) or genitourinary system and sex hormones (three participants; 8%).

**TABLE 1:** Summary of demographic and baseline characteristics by BAL sampling time point group

Characteristic	30 minute BAL (*N* = 7)^*a*^	75 minute BAL (*N* = 6)	2 hour BAL (*N* = 7)	5 hour BAL (*N* = 13)	8 hour BAL (*N* = 6)	All participants (*N* = 39)
Sex, n (%)						
Male	4 (57)	5 (83)	3 (43)	6 (46)	3 (50)	21 (54)
Female	3 (43)	1 (17)	4 (57)	7 (54)	3 (50)	18 (46)
Ethnicity, n (%)						
Not Hispanic or Latino	6 (86)	6 (100)	6 (86)	10 (77)	6 (100)	34 (87)
Hispanic or Latino	1 (14)	-^*b*^	1 (14)	2 (15)	-	4 (10)
Not reported	-	-	-	1 (8)	-	1 (3)
Unknown	-	-	-	-	-	-
Race, n (%)						
American Indian or Alaska Native	-	1 (17)	-	-	-	1 (3)
Asian	2 (29)	1 (17)	1 (14)	1 (8)	-	5 (13)
Native Hawaiian or Other Pacific Islander	-	-	-	-	-	-
Black or African American	2 (29)	2 (33)	4 (57)	5 (38)	2 (33)	15 (38)
White	3 (43)	2 (33)	2 (29)	5 (38)	4 (67)	16 (41)
Multiracial	-	-	-	1 (8)	-	1 (3)
Other	-	-	-	1 (8)	-	1 (3)
Age (years)						
Mean (SD)	31.3 (6.5)	31.2 (4.6)	31.4 (6.8)	32.2 (6.6)	30.3 (9.4)	31.4 (6.5)
Median	32.0	31.0	30.0	31.0	27.5	30.0
Minimum; maximum	24, 43	25, 38	22, 40	19, 43	20, 45	19, 45
Height (cm)						
Mean (SD)	170.26 (10.52)	173.00 (8.67)	163.07 (4.54)	167.35 (8.66)	173.28 (8.17)	168.89 (8.73)
Median	167.50	176.30	164.00	165.50	170.90	167.30
Minimum; maximum	158.0, 183.0	160.4, 182.0	156.5, 168.6	156.0, 182.7	166.8, 189.5	156.0, 189.5
Weight (kg)						
Mean (SD)	70.64 (11.87)	73.98 (11.26)	67.60 (6.14)	74.96 (12.68)	75.78 (7.49)	72.84 (10.59)
Median	76.20	70.40	69.50	77.20	77.50	72.50
Minimum; maximum	50.5, 83.2	61.3, 88.5	55.0, 73.6	57.0, 94.3	63.9, 83.2	50.5, 94.3
BMI (kg/m^2^)						
Mean (SD)	24.31 (3.35)	24.60 (2.11)	25.47 (2.68)	26.62 (3.02)	25.27 (2.40)	25.48 (2.82)
Median	24.60	24.15	25.30	26.70	25.60	25.30
Minimum; maximum	18.8, 29.8	22.1, 27.5	20.2, 28.6	21.8, 30.4	22.0, 28.0	18.8, 30.4

^*a*^ *N*, number of participants in the safety population.

^*b*^ -, not represented in study population.

### Pharmacokinetic analysis

All 39 participants (100%) received the first and second dose of fosfomycin. Thirty-seven participants (95%) received Dose 3. Thirty participants (77%) completed bronchoscopy with an accompanying plasma pharmacokinetic sample. Nine participants did not complete all three doses of the study product and/or did not undergo BAL at the assigned sampling time point, and thus, were excluded from the PK analysis population.

Mean plasma fosfomycin concentrations at 0.5 hour, 1 hour, 1.25 hour, 2 hour, 5 hour, and 8 hour after dose 1, respectively, were 196.4 µg/mL, 297.4 µg/mL, 247.5 µg/mL, 156.2 µg/mL, 51.5 µg/mL, and 23.6 µg/mL, respectively. Mean plasma fosfomycin concentrations at 0.5 hour, 1 hour, 1.25 hour, 2 hour, 5 hour, 8 hour, and 12 hour after dose 3, respectively, were 234.6 µg/mL, 335.1 µg/mL, 269.6 µg/mL, 190.7 µg/mL, 71.5 µg/mL, 32.4 µg/mL, and 12.0 µg/mL, respectively. The median peak time (T_max_) in plasma was 1.05 hour after the start of dose 1 and 1.06 hour after the start of dose 3. The geometric mean (GM) of the observed accumulation ratios (ARs) comparing doses 1 and 3 for AUC_(0-8)_ was 1.2, and the geometric standard deviation (GSD) was 1.128. The GM of the AR for the maximum measured plasma concentration (C_max_) was 1.122, and the GSD was 1.172.

In the non-compartmental PK analysis of plasma concentration, the GM of C_max_ after dose 1 was 293.9 µg/mL with a coefficient of variation as a percentage (CV%) of 16%. The GM of C_max_ after dose 3 was 329.6 µg/mL with a CV% of 18%. The GM of AUC_(0-8)_ was 948.1 h* µg/mL with a CV% of 16%. The GM of AUC _(0-inf)_ was 1070.5 h* µg/mL with a CV% of 18%. The GM for half-life (t_1/2_) was 2.59 hours with a CV% of 14%.

Two ELF aliquots were analyzed for all participants. If the two ELF concentrations were within 20% of each other, the average ELF concentration was reported. For six pairs of aliquots that differed by 20%–40%, the two concentration results were averaged. One pair of aliquots differed by 57%, and this participant’s data were excluded from the final PK analyses of fosfomycin concentration in ELF. This was based on FDA guidance for repeat analysis and incurred sample reanalysis (Bioanalytical Method Validation [2018] and FDA guidance [2022] M10 Bioanalytical Method Validation and Study Sample Analysis [https://www.fda.gov/media/162903/download]).

**Fig 2:**
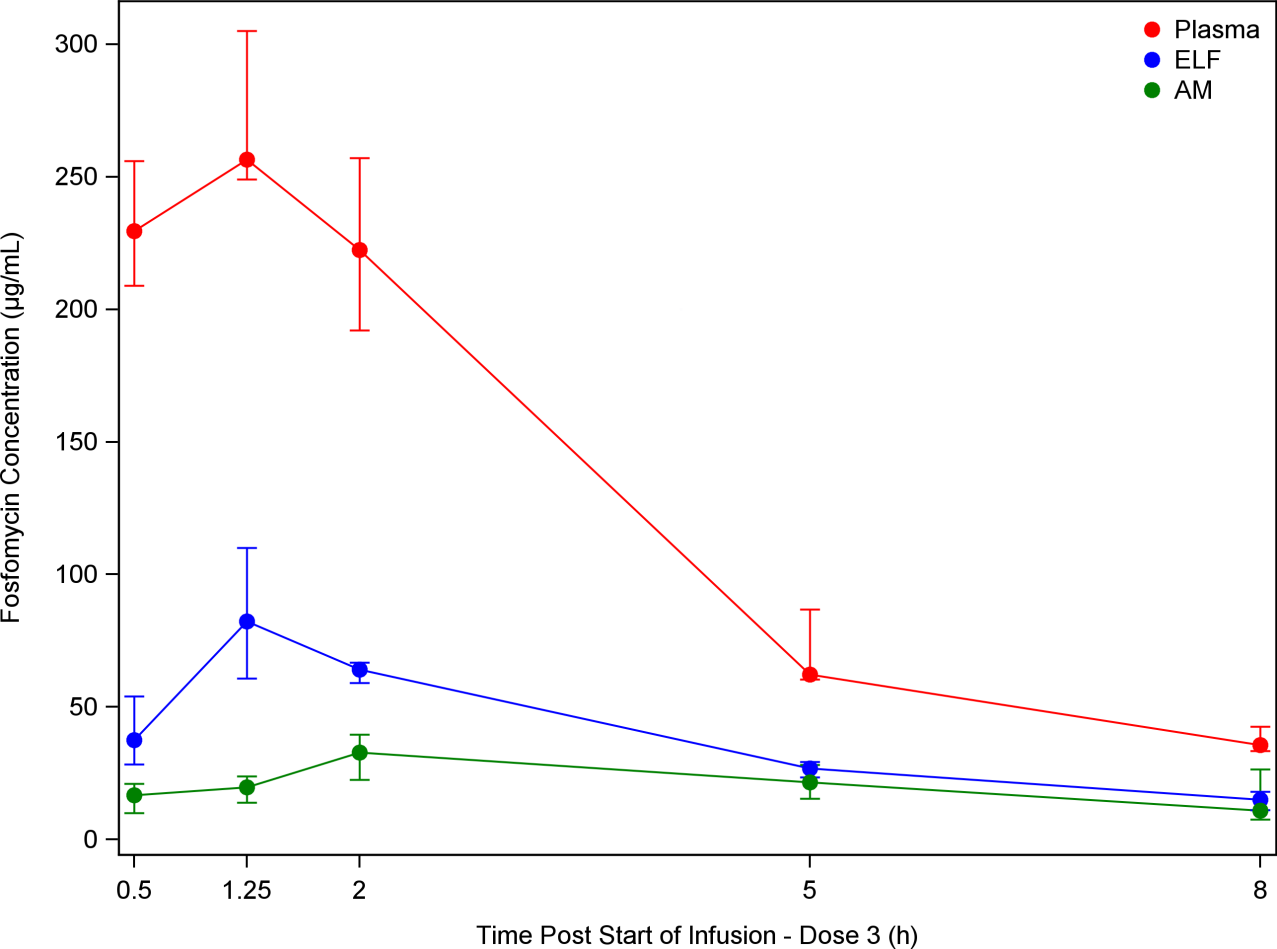
Semi-log plot of population median (intraquartile range) fosfomycin concentrations in plasma, ELF, and AM by time (h) after start of dose 3.

The GM concentrations of fosfomycin in ELF at 0.5 hour, 1.25 hour, 2 hour, 5 hour, and 8 hour after dose 3 were 42.2 µg/mL, 78.2 µg/mL, 63.4 µg/mL, 25.1 µg/mL, and 15.0 µg/mL, respectively, and, in AM, were 17.0 µg/mL, 19.4 µg/mL, 30.3 µg/mL, 20.0 µg/mL, and 12.6 µg/mL, respectively. Hence, the peak concentration in ELF occurred at 1.25 hours after the start of dose 3, while the peak concentration in AM occurred slightly later, at 2 hours after the start of dose 3. Figure 2 demonstrates median fosfomycin concentration–time profiles after the start of dose 3 in plasma, ELF, and AM.

The results of ELF and AM fosfomycin concentrations and the results of ELF- and AM-to-plasma concentration ratio by BAL sampling time point are summarized in Table 2 (ELF) and Table 3 (AM), showing the trend of increased penetration with time, and up to 42% and 43% for ELF and AM at 8 hours after dosing, respectively. The results of the calculated percent lung penetration for ELF and AM by average AUC_(0-8)_ are summarized in Table 4. Using the median concentrations at the BAL sampling time points, the percent lung penetration for ELF was estimated to be 31.8% and the percent lung penetration for AM was estimated to be 17.5%. Using the GM concentrations at the BAL sampling time points, the percent lung penetration for ELF was estimated to be 30.8%, and the percent lung penetration for AM was estimated to be 16.8%.

**TABLE 2:** Summary statistics for ELF fosfomycin concentration (µg/mL) and ELF to plasma concentration ratio by BAL sampling timepoint^*a*^

	ELF fosfomycin concentration (µg/mL) at BAL sampling timepoint					Concentration ratio ELF to plasma at BAL sampling time point				
Statistic	0.5 h	1.25 h	2 h	5 h	8 h	0.5 h	1.25 h	2 h	5 h	8 h
*N*	6	5	6	6	6	6	5	6	6	6
Mean	47.6	82.5	63.6	27.0	15.7	0.20	0.32	0.29	0.40	0.42
SD	28.7	28.9	6.3	10.5	5.2	0.10	0.097	0.050	0.075	0.12
Min	26.0	47.4	54.8	11.5	10.8	0.12	0.19	0.24	0.30	0.30
Median	37.6	82.2	64.1	26.9	15.0	0.16	0.32	0.28	0.39	0.40
Max	102.4	112.1	72.8	43.7	24.5	0.40	0.45	0.37	0.50	0.57
CV%	54	39	10	46	32	47	33	17	19	28
GM	42.2	78.2	63.4	25.1	15.0	0.18	0.30	0.29	0.39	0.41
GSD	1.7	1.5	1.1	1.6	1.4	1.6	1.4	1.2	1.2	1.3

^*a*^ Time points are relative to time of start of infusion of Dose 3; *N*, number of data points used to compute the summary statistics.

**TABLE 3:** Summary statistics for AM fosfomycin concentration (μg/mL) and AM to plasma concentration ratio by BAL sampling timepoint^*a*^

	AM fosfomycin concentration (µg/mL) at BAL sampling time point					Concentration ratio AM to plasma at BAL sampling time point				
Statistic	0.5 h	1.25 h	2 h	5 h	8 h	0.5 h	1.25 h	2 h	5 h	8 h
*N*	6	6	6	6	6	6	6	6	6	6
Mean	19.5	20.7	32.0	21.6	14.8	0.084	0.078	0.14	0.33	0.43
SD	12.3	8.2	11.1	8.5	9.3	0.055	0.024	0.034	0.12	0.31
Min	9.4	12.6	17.2	9.6	6.4	0.031	0.053	0.098	0.24	0.15
Median	16.8	19.7	32.7	21.6	10.8	0.069	0.077	0.14	0.29	0.31
Max	43.0	34.4	47.5	33.4	26.8	0.19	0.11	0.18	0.54	0.88
CV%	60	40	39	47	68	67	31	25	32	86
GM	17.0	19.4	30.3	20.0	12.6	0.072	0.075	0.14	0.31	0.34
GSD	1.7	1.5	1.5	1.6	1.8	1.8	1.4	1.3	1.4	2.1

^*a*^ Time points are relative to time of start of infusion of Dose 3. *N*, number of data points used to compute the summary statistics.

**TABLE 4:** Average AUC_0-8_ of fosfomycin in plasma, ELF, and AM and percent penetration

Estimate type	Sample	*N^a^*	AUC_(0-8)_^*b*^ (h*µg/mL)	Percent penetration (%)
Median^*c*^	Plasma	30	939.8	NA^*e*^
	ELF	29	298.4	31.8
	AM	30	164.8	17.5
Mean^*d*^	Plasma	30	947.5	NA
	ELF	29	291.4	30.8
	AM	30	159.2	16.8

^*a*^ *N*, number of data points used to compute AUC_(0-8)_.

^*b*^ AUC_(0-8)_ following Dose 3.

^*c*^ The median concentrations at each BAL time point were used to compute the population average AUC_(0-8)_.

^*d*^ The geometric mean concentrations at each BAL time point were used to compute the population average AUC_(0-8)_.

^*e*^ NA, not applicable.

### Safety analysis

All participants (100%) experienced at least one TEAE. No participant experienced an SAE. Thirty-eight participants (97%) experienced treatment-related TEAEs of the following grades of severity: mild/grade 1 (36 participants), moderate/grade 2 (12 participants), and severe/grade 3 (2 participants). All symptomatic TEAEs resolved or stabilized before the end of the study. Sixteen TEAEs, the majority of which represented mild, asymptomatic lab abnormalities, were not able to be confirmed as resolved due to participants not returning for additional specimen collection.

TEAEs are presented in Table S2. Participants had TEAEs in the following categories: lab abnormalities, 37 participants (95%); cardiac disorders, 15 participants (38%); respiratory, thoracic, and mediastinal disorders, 14 participants (36%); general disorders and administration site conditions, 10 participants (26%); GI disorders, nine participants (23%); vascular disorders, seven participants (18%); nervous system disorders, five participants (13%); skin and subcutaneous tissue disorders, two participants (5%); musculoskeletal and connective tissue disorders, one participant (3%); and renal and urinary disorders, one participant (3%). Of the TEAEs involving lab abnormalities, electrolyte abnormalities, specifically, included hypomagnesemia in 29 (74%) participants, all of mild severity; hypokalemia in 19 (49%) participants, all of mild severity; and hypocalcemia in 16 (41%) participants, with 11 of those (28%) being mild and 5 (13%) of moderate severity.

There were two severe TEAEs: one participant (3%) experienced hypophosphatemia, which was determined to be related to the study treatment, and one participant (3%) experienced fatigue, which was not determined to be related to the study treatment, but rather, to longer than the expected effect of the sedation used for the bronchoscopy procedure.

A total of eight participants (21%) were withdrawn due to moderate or severe TEAEs, all considered treatment-related: moderate or severe hypophosphatemia (two participants, 5%), moderate hypocalcemia (four participants, 10%), and moderate nausea and/or vomiting (two participants, 5%).

## DISCUSSION

This is the first investigation to evaluate the penetration of fosfomycin into the ELF and AM and to derive its pulmonary pharmacokinetics. The plasma PK parameters observed in this study for fosfomycin were consistent with those previously reported in healthy participants (15). Plasma PK exposures (AUC and C_max_) were similar after dose 1 and dose 3 of IV fosfomycin, with less than 20% accumulation. Peak time was also no different, and t_1/2_ was approximately 3 hours.

The concentrations of fosfomycin in ELF and AM were examined at five different points throughout the dosing period, allowing for the creation of a pulmonary PK profile. The peak concentration of fosfomycin in ELF was 78.2 µg/mL at 1.25 hour, and the peak concentration in AM was 30.3 µg/mL at 2 hours. The median AUC_(0-8)_ values were 298.4 h* µg/mL for ELF and 164.8 h* µg/mL for AM.

The percent penetration values for fosfomycin from plasma into ELF and AM were 30.8% and 16.8%, respectively, using the geometric mean concentrations from the BAL sampling time points and were 31.8% and 17.5%, respectively, using the median concentrations from the BAL sampling time points. This is consistent with prior data showing the fosfomycin is predominantly distributed in the ELF (approximately 0.30 L/kg body weight). The peak concentration in ELF was noted at 1.25 hours, while the peak concentration in AM was noted slightly later, at 2 hours. There was a trend of increased penetration over time, with up to 42% and 43% for ELF and AM at 8 hours after dosing, respectively.

This finding is similar to the finding in the two prior studies that focused on fosfomycin penetration into lung tissue in subjects undergoing surgical lung resection. Farago et al*.* reported concentrations, in blocks of resected lung tissue, of 32%–52% of corresponding serum concentrations when measured 1–2 hours after a single 2-g dose of IV fosfomycin (9). Matzi *et al.* used microdialysis probes into the extracellular space to report interstitial lung concentrations of 44%–55% of corresponding serum concentrations, when measured 1 hour after a single 4-g dose of IV fosfomycin (4). The method of fosomycin measurement in both of those prior studies differed substantially from this study, which was able to differentiate ELF and AM measurements and to eliminate inclusion of blood in the specimens obtained, which would be expected to falsely increase the concentrations measured.

The concentrations of fosfomycin achieved in ELF and AM suggest that fosfomycin may be an appropriate antibiotic for the treatment of many common organisms causing hospital-acquired and ventilator-associated pneumonia, and as part of a combination regimen, including additional coverage for atypical organisms, for community-acquired pneumonia (16). The mean AM fosfomycin concentrations ranged from 14.8 to 32.0 µg/mL, and the mean ELF concentration of fosfomycin ranged from 15.7 to 82.5 µg/mL. These concentrations may be effective against *Staphylococcus aureus* including both MSSA (MIC50 4 µg/mL; MIC90 4 µg/mL) and some MRSA (MIC50 4 µg/mL; MIC90 8–64 µg/mL) strains. Fosfomycin is also likely to be effective against some Gram-negative bacteria such as ESBL-producing and AmpC-producing *E. coli* (MIC50 0.5–4 µg/mL; MIC90 1–16 µg/mL) and *Haemophilus* spp. (MIC50 1 µg/mL; MIC90 4 µg/mL) and other enteric Gram-negative bacteria such as *Klebsiella oxytoca* (MIC50 8; MIC90 16–32 µg/mL), some *Klebsiella pneumoniae* (MIC50 4–16 µg/mL; MIC90 16–128 µg/mL), and *Serratia* spp. (MIC50 8 µg/mL, MIC90 16–32 µg/mL). However, the concentrations of fosfomycin achieved in ELF and BAL, with this dosing regimen, may not be high enough to use as a sole agent to treat pneumonias caused by *Pseudomonas aeruginosa* (MIC50 32–64 µg/mL; MIC90 64–128 µg/mL) and *Stenotrophomonas maltophilia* (MIC50 64–128 µg/mL; MIC90 128 µg/mL) (17).

Overall, IV fosfomycin was well tolerated. Most participants (97%) experienced at least one treatment-related TEAE, but these were largely mild/grade 1 (92%). Of the 31% moderate/grade 2 TEAEs, most (21%) were asymptomatic laboratory abnormalities, with the remaining 10% being gastrointestinal side effects. Only one participant (3%) experienced a severe TEAE related to the study treatment, hypophosphatemia, and one participant (3%) experienced an unrelated severe TEAE, fatigue, which was related to the sedation for the bronchoscopy procedure, not to the study treatment. There were no serious adverse events or deaths in this study.

Side effects previously reported to be frequently associated with IV fosfomycin have included asymptomatic electrolyte abnormalities such as hypokalemia, hypocalcemia, and hypophosphatemia, gastrointestinal side effects, mild reductions in heart rate, and mild headache (11). In this trial, all of hypokalemia cases were mild, but did occur in 49% of participants. Hypocalcemia occurred in 41% of participants, which was mild in 28% and moderate in 13%. The occurrence of moderate and severe hypophosphatemia in three participants (8%) was asymptomatic, but led to early termination in two participants due to the degree of derangement being moderate or severe. Hypomagnesemia was also frequent, but exclusively mild, occurring in 74% of participants. One participant had a moderately decreased neutrophil count, which has also previously been reported with IV fosfomycin. Gastrointestinal side effects occurred in 18% of participants, all of mild or moderate severity. Bradycardia, which had an incidence of up to 29% in a prior study of a higher dose of IV fosfomycin (11), did not occur in this study. Elevations in hepatic transaminases, also seen in a prior study of IV fosfomycin (10), also did not occur in this study.

An important limitation of this study is that it was conducted in healthy, young participants, aged 18–45, with no acute or chronic illnesses, under fasting conditions, and who were on no significant concomitant medications. Thus, the pharmacokinetic and safety findings observed may not be generalizable to patients who are critically ill, potentially with both pulmonary and extrapulmonary organ dysfunction, and likely receiving multiple concomitant medications. Additionally, this study included only three doses, whereas complete treatment durations of multiple days could result in higher frequency or severity of side effects. Specifically, if the 18% rate of nausea and vomiting seen in this single-day dosing period was to increase with a longer duration of therapy, this could be a treatment-limiting side effect. A higher incidence of asymptomatic lab abnormalities over a longer duration of treatment could be mitigated by close monitoring within an inpatient setting.

However, the rapid achievement of peak fosfomycin concentrations (1.06 hour in plasma, 1.25 hour in ELF, and 2 hour in AM cells) indicated rapid pulmonary penetration after the start of the third dose of fosfomycin. This may prove especially important in critically ill patients, in whom it is vital to rapidly achieve sufficient antibiotic concentrations at the site of infection. The significant and prolonged levels of fosfomycin in plasma, ELF, and AM lead to the conclusion that IV fosfomycin may be an effective antibacterial treatment for lower respiratory tract bacterial infections that are caused by susceptible pathogens.

## SUPPLEMENTAL MATERIAL

Supplemental material aac.01395-24-s0001.docx Tables S1 and S2; Figure S1.

ASM does not own the copyrights to Supplemental Material that may be linked to, or accessed through, an article. The authors have granted ASM a non-exclusive, world-wide license to publish the Supplemental Material files. Please contact the corresponding author directly for reuse.
