# Supplementary material for: Evaluation of the safety profile and intrapulmonary pharmacokinetics of intravenous fosfomycin in healthy adults
Source: Antimicrob Agents Chemother. 2025 Jan 8;69(2):e01395-24. doi: 10.1128/aac.01395-24 (PMC11823650; doi:10.1128/aac.01395-24)
Supplement: Supplemental material — Tables S1 and S2; Figure S1. [file aac.01395-24-s0001.docx]

**Appendix Table 1: Inclusion and Exclusion Criteria**

| **Inclusion Criteria** | **Exclusion Criteria** |
| --- | --- |
| Age 18-45 year | Medical condition that could place the participant at increased risk while participating in the trial, prevent a participant from undergoing bronchoscopy |
| Absence of clinically significant findings on screening and baseline evaluations | Medical condition that could interfere with drug absorption, distribution, metabolism, or excretion |
| Body weight >50 kg with body mass index 18-30 kg/m^2^ | Cardiac impairment or clinically significant ECG abnormality |
| Willingness to use acceptable contraception | Renal impairment (Cr >1.0 mg/dL for females or >1.3 mg/dL for males) |
| Willingness to abstain from caffeine for 7 days prior to confinement | Liver impairment, including viral hepatitis |
| Willingness to comply with confinement and all study procedures | Pulmonary impairment |
|  | HIV |
|  | Cancer or recent history of cancer (past 5 years), other than non-melanomatous skin cancer |
|  | Anemia (Hb<11.8 g/dL for females or <13.7 g/dL for males) |
|  | Screening lab value outside acceptable range, considered by the investigators to be clinically significant |
|  | COVID-19 infection within the last 6 weeks, or positive COVID-19 test at screening or enrollment |
|  | Loss or gain of >10% body weight within 30 days |
|  | Use of certain prescription or non-prescription drugs, including caffeine and excess licorice |
|  | History of excessive alcohol consumption in the prior 6 months (>14 drinks/week for males or >7 drinks/week for females) |
|  | History of ≥10 pack-years smoking or any nicotine use within the prior 6 months |
|  | Illicit drug use within the prior 5 months |
|  | Pregnancy or breastfeeding |
|  | Recent blood donation |
|  | Planned egg or sperm donation during the study |

Appendix Figure 1: Schematic of Study Design


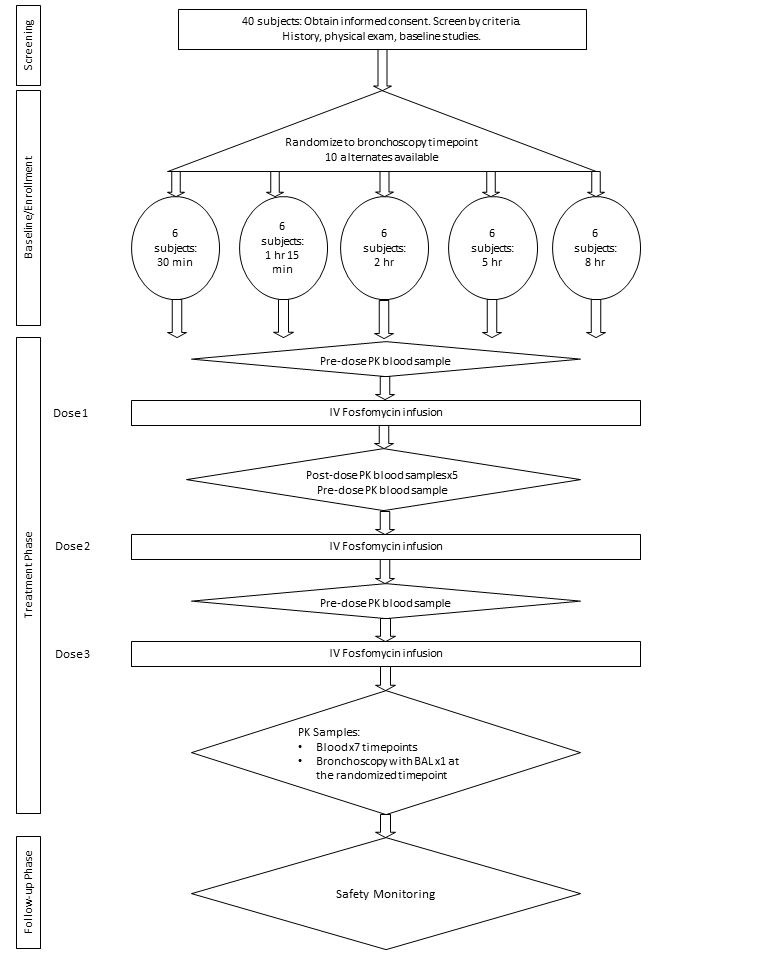


Appendix Table 2: Unsolicited Adverse Events by MedDRA System Organ Class and Preferred Term, Maximum Severity and Relationship

|  |  |  | **All Participants (N = 39)** | | | |
| --- | --- | --- | --- | --- | --- | --- |
|  |  |  | **Related** | | **Not Related** | |
| **MedDRA System Organ Class** | **Preferred Term** | **Severity** | **n** | **%** | **n** | **%** |
| Any SOC | Any PT | Any Severity | 38 | 97 | 31 | 79 |
|  |  | Mild | 25 | 64 | 24 | 62 |
|  |  | Moderate | 12 | 31 | 6 | 15 |
|  |  | Severe | 1 | 3 | 1 | 3 |
| Cardiac disorders | Any PT | Any Severity | 1 | 3 | 14 | 36 |
|  |  | Mild | 1 | 3 | 10 | 26 |
|  |  | Moderate | - | - | 4 | 10 |
|  |  | Severe | - | - | - | - |
|  | Bradycardia | Any Severity | - | - | 7 | 18 |
|  |  | Mild | - | - | 6 | 15 |
|  |  | Moderate | - | - | 1 | 3 |
|  |  | Severe | - | - | - | - |
|  | Sinus bradycardia | Any Severity | - | - | 1 | 3 |
|  |  | Mild | - | - | 1 | 3 |
|  |  | Moderate | - | - | - | - |
|  |  | Severe | - | - | - | - |
|  | Sinus tachycardia | Any Severity | - | - | 4 | 10 |
|  |  | Mild | - | - | 2 | 5 |
|  |  | Moderate | - | - | 2 | 5 |
|  |  | Severe | - | - | - | - |
|  | Tachycardia | Any Severity | 1 | 3 | 3 | 8 |
|  |  | Mild | 1 | 3 | 2 | 5 |
|  |  | Moderate | - | - | 1 | 3 |
|  |  | Severe | - | - | - | - |
| Gastrointestinal disorders | Any PT | Any Severity | 7 | 18 | 2 | 5 |
|  |  | Mild | 3 | 8 | 1 | 3 |
|  |  | Moderate | 4 | 10 | 1 | 3 |
|  |  | Severe | - | - | - | - |
|  | Abdominal pain | Any Severity | 1 | 3 | 2 | 5 |
|  |  | Mild | - | - | 1 | 3 |
|  |  | Moderate | 1 | 3 | 1 | 3 |
|  |  | Severe | - | - | - | - |
|  | Nausea | Any Severity | 4 | 10 | - | - |
|  |  | Mild | 3 | 8 | - | - |
|  |  | Moderate | 1 | 3 | - | - |
|  |  | Severe | - | - | - | - |
|  | Vomiting | Any Severity | 4 | 10 | - | - |
|  |  | Mild | 2 | 5 | - | - |
|  |  | Moderate | 2 | 5 | - | - |
|  |  | Severe | - | - | - | - |
| General disorders and administration site conditions | Any PT | Any Severity | 3 | 8 | 7 | 18 |
|  |  | Mild | 3 | 8 | 6 | 15 |
|  |  | Moderate | - | - | - | - |
|  |  | Severe | - | - | 1 | 3 |
|  | Asthenia | Any Severity | 1 | 3 | - | - |
|  |  | Mild | 1 | 3 | - | - |
|  |  | Moderate | - | - | - | - |
|  |  | Severe | - | - | - | - |
|  | Chest discomfort | Any Severity | - | - | 1 | 3 |
|  |  | Mild | - | - | 1 | 3 |
|  |  | Moderate | - | - | - | - |
|  |  | Severe | - | - | - | - |
|  | Fatigue | Any Severity | - | - | 1 | 3 |
|  |  | Mild | - | - | - | - |
|  |  | Moderate | - | - | - | - |
|  |  | Severe | - | - | 1 | 3 |
| General disorders and administration site conditions | Infusion site erythema | Any Severity | 1 | 3 | - | - |
|  |  | Mild | 1 | 3 | - | - |
|  |  | Moderate | - | - | - | - |
|  |  | Severe | - | - | - | - |
|  | Infusion site irritation | Any Severity | 1 | 3 | - | - |
|  |  | Mild | 1 | 3 | - | - |
|  |  | Moderate | - | - | - | - |
|  |  | Severe | - | - | - | - |
|  | Infusion site pain | Any Severity | - | - | 3 | 8 |
|  |  | Mild | - | - | 3 | 8 |
|  |  | Moderate | - | - | - | - |
|  |  | Severe | - | - | - | - |
|  | Infusion site swelling | Any Severity | - | - | 1 | 3 |
|  |  | Mild | - | - | 1 | 3 |
|  |  | Moderate | - | - | - | - |
|  |  | Severe | - | - | - | - |
|  | Vessel puncture site pain | Any Severity | - | - | 2 | 5 |
|  |  | Mild | - | - | 2 | 5 |
|  |  | Moderate | - | - | - | - |
|  |  | Severe | - | - | - | - |
| Investigations | Any PT | Any Severity | 36 | 92 | 7 | 18 |
|  |  | Mild | 27 | 69 | 7 | 18 |
|  |  | Moderate | 8 | 21 | - | - |
|  |  | Severe | 1 | 3 | - | - |
|  | Blood albumin decreased | Any Severity | 3 | 8 | - | - |
|  |  | Mild | 3 | 8 | - | - |
|  |  | Moderate | - | - | - | - |
|  |  | Severe | - | - | - | - |
|  | Blood calcium decreased | Any Severity | 16 | 41 | - | - |
|  |  | Mild | 11 | 28 | - | - |
|  |  | Moderate | 5 | 13 | - | - |
|  |  | Severe | - | - | - | - |
| Investigations | Blood creatine phosphokinase increased | Any Severity | 1 | 3 | - | - |
|  |  | Mild | 1 | 3 | - | - |
|  |  | Moderate | - | - | - | - |
|  |  | Severe | - | - | - | - |
|  | Blood glucose increased | Any Severity | 1 | 3 | 6 | 15 |
|  |  | Mild | 1 | 3 | 6 | 15 |
|  |  | Moderate | - | - | - | - |
|  |  | Severe | - | - | - | - |
|  | Blood magnesium decreased | Any Severity | 29 | 74 | - | - |
|  |  | Mild | 29 | 74 | - | - |
|  |  | Moderate | - | - | - | - |
|  |  | Severe | - | - | - | - |
|  | Blood phosphorus decreased | Any Severity | 4 | 10 | - | - |
|  |  | Mild | 1 | 3 | - | - |
|  |  | Moderate | 2 | 5 | - | - |
|  |  | Severe | 1 | 3 | - | - |
|  | Blood potassium decreased | Any Severity | 19 | 49 | - | - |
|  |  | Mild | 19 | 49 | - | - |
|  |  | Moderate | - | - | - | - |
|  |  | Severe | - | - | - | - |
|  | Blood sodium decreased | Any Severity | 1 | 3 | - | - |
|  |  | Mild | 1 | 3 | - | - |
|  |  | Moderate | - | - | - | - |
|  |  | Severe | - | - | - | - |
|  | Blood sodium increased | Any Severity | 1 | 3 | - | - |
|  |  | Mild | 1 | 3 | - | - |
|  |  | Moderate | - | - | - | - |
|  |  | Severe | - | - | - | - |
|  | Electrocardiogram QT prolonged | Any Severity | 1 | 3 | - | - |
|  |  | Mild | 1 | 3 | - | - |
|  |  | Moderate | - | - | - | - |
|  |  | Severe | - | - | - | - |
